# Dissection of the Sympathetic Nerves Around the Mesorectum at the Abdominopelvic Border

**DOI:** 10.7759/cureus.69091

**Published:** 2024-09-10

**Authors:** Theodor G Badea, Iulian A Dogaru, Zoran F Filipoiu, Daniela E Gheoca Mutu, Florin Filipoiu

**Affiliations:** 1 Medicine, Doctoral School, Carol Davila University of Medicine and Pharmacy, Bucharest, ROU; 2 Radiology, Prof. Dr. Agrippa Ionescu Clinical Emergency Hospital, Bucharest, ROU; 3 General Surgery, Prof. Dr. Agrippa Ionescu Clinical Emergency Hospital, Bucharest, ROU; 4 Medicine, Carol Davila University of Medicine and Pharmacy, Bucharest, ROU; 5 Anatomy, Carol Davila University of Medicine and Pharmacy, Bucharest, ROU; 6 Plastic and Reconstructive Surgery, Prof. Dr. Agrippa Ionescu Clinical Emergency Hospital, Bucharest, ROU

**Keywords:** erector nerves, inferior hypogastric plexus, mesorectum, pelvic anatomy, pelvic innervation, rectal resection, sacral splanchnic nerves, superior hypogastric plexus

## Abstract

Introduction

Along the border between the abdominal cavity and pelvis are nervous structures that belong to the autonomous nervous system, which is delicate. These can be easily injured during regional surgical procedures such as the total mesorectal excision, where the preservation of the nervous structures should be one of the main objectives. In our study, we aimed to dissect all the sympathetic nerve formations listed at the abdominopelvic border and to present their formation, anatomical routes, and relations, as well as the surgical importance of their preservation.

Method

We performed anatomical dissections on eight 60- to 75-year-old cadavers (three male and five female) in the Dissection Laboratory of Carol Davila University of Medicine and Pharmacy, Bucharest, ROM. We sectioned each pelvis along the right pararectal line and exposed the hypogastric plexuses and their branches, following their pathways toward the pelvic viscera.

Results

We highlight the main nervous structures in the pelvis, namely the paravertebral sympathetic ganglion chain, which continues into the pelvis with the sacral ganglion chain, and the prevertebral component of the abdominal sympathetic system, represented by the superior hypogastric plexus and its continuation via the hypogastric nerves toward the inferior hypogastric plexuses. We followed the pathway of the superior hypogastric plexus from its origin down to its bifurcation into the two hypogastric nerves. We then followed the nerves into the pelvis and observed the formation of the inferior hypogastric plexuses, from which branches emerged toward the pelvic organs. Along the way, we point out anatomical landmarks that are crucial in an attempt to spare these nervous structures during regional surgical procedures.

Conclusions

While performing surgeries such as rectal resection with the excision of the mesorectum, radical hysterectomy, and radical prostatectomy, a thorough knowledge of the sympathetic nerve structures that pass from the abdominal cavity into the pelvis is required to spare pelvic innervation. In such a context, the dissection and anatomical assessment of regional sympathetic nerves can prove to be crucial in establishing operative protocols.

## Introduction

During fetal development, the primitive dorsal mesentery connects the primitive intestinal tract to the posterior wall of the trunk, extending down to the level of the anal canal [1]. Gradually, the dorsal mesentery disappears during coalescence, and the posterior parietal peritoneum forms the pararectal peritoneal recesses in the pelvis. At the level of the rectosigmoid junction, the digestive tract turns from entirely intraperitoneal into partially intraperitoneal. The retrorectal space is delimitated anteriorly by the rectum and is located between the rectum and the sacrum [2]. This space contains the presacral fascia, the anterior sacral foramina (through which the sacral spinal nerves and their branches emerge), and the sacral sympathetic chains (which merge inferiorly at the level of a single coccygeal ganglion) [3]. This space also contains the presacral artery, presacral lymph nodes, and a mass of loose connective tissue, which, if properly tractioned intraoperatively, gives the aspect of “angel's hair” [4]. This loose connective tissue develops as a result of rectal coalescence during the disappearance of the caudal part of the primitive mesentery [5]. Around the rectum, the perirectal fascia (rectal sheath according to Thoma Ionescu [6,7]) is differentiated. Between the periectal fascia and the rectum lies the mesorectal space, or the mesorectum [8]. It contains the upper rectal vessels, the upper rectal lymph nodes, and the connective-adipose tissue. En-bloc resection of the rectum and the mesorectum represents, nowadays, a golden standard in modern rectal cancer surgery [9,10].

This standard has been ultimately improved by nerve-sparing surgery [11,12], which implies the protection of the nerve structures described above, as well as the preservation of the superior hypogastric plexus and the two hypogastric nerves, which follow a laterorectal, subperitoneal route to the pelvic subperitoneal compartment [13], where they constitute the inferior hypogastric plexus [14]. Of utmost importance is also the protection of the pelvic splanchnic (erector) nerves, which are vital in maintaining sexual function [15,16]. In our study, we aimed to dissect all the sympathetic nerve formations found at the abdominopelvic border and present their formation and anatomical routes and relations, as well as the surgical importance of their preservation.

## Materials and methods

The dissection study was conducted on eight cadavers aged between 60 and 75 years (three males and five females). The cadavers for this study had no previous medical or surgical history. The cadavers were preserved with a 9% formalin solution in the dissection laboratory of the Department of Anatomy at Carol Davila University of Medicine and Pharmacy, Bucharest, Romania. The dissections were performed in compliance with the national laws regarding the manipulation of cadavers and university regulations (Law No. 104/27.03.2003).

Each pelvis was sectioned along the right pararectal plane. In the presacral compartment and on the lateral wall of the pelvis, we performed a highly detailed anatomical dissection of the main structures found in the pelvisubperitoneal space. During the dissection, we exposed the hypogastric plexuses and their branches. We removed the pelvic peritoneum for each hemipelvis and, after identifying the hypogastric nerves, applied traction on them to facilitate the dissection of the inferior hypogastric plexus. The left hypogastric nerve was detached from the pelvic floor and drawn superiorly to highlight the sacral sympathetic chain, the inferior hypogastric plexus, and the anastomoses between them (the pelvic splanchnic nerves). We then traced the branches of the inferior hypogastric plexus towards the pelvic organs.

The results of our carefully conducted dissection were photographed, focusing on the areas surrounding the mesorectum. In our dissection images, the inferior hypogastric plexus has been detached from the pelvic floor and folded medially, towards the rectum. Some branches of the internal iliac artery and the ureter were preserved to better observe their intricate relations with the nervous structures. All images were digitally enhanced for better clarity. No alterations have been made in what concerns the scientific content.

## Results

**Figure 1:**
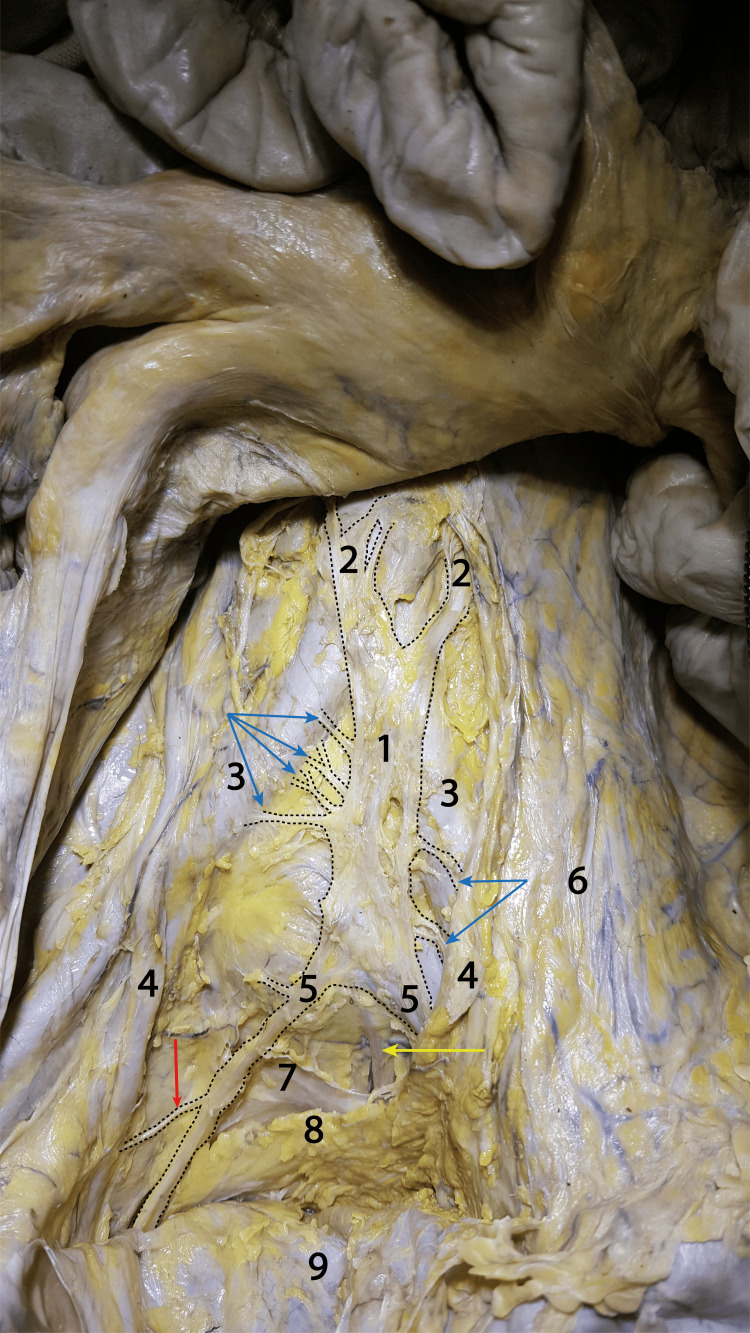
Dissection revealing the formation of the superior hypogastric plexus and the hypogastric nerves Red arrow: Ureteral nervous branches; Yellow arrow: Presacral vascular bundle; Blue arrows: Common iliac periarterial branches from the superior hypogastric plexus; 1: Superior hypogastric plexus; 2: Origin fascicles of the superior hypogastric plexus; 3: Common iliac arteries (right and left); 4: Right and left ureters; 5: Right and left hypogastric nerves; 6: Posterior parietal peritoneum; 7: Presacral fascia; 8: Neuroconnective lamina between the hypogastric nerves; 9: The rectum tractioned anteriorly

Our dissected cadavers did not present any significant abnormalities. The only notable occurrence was the trajectory of the abdominal aorta in one of the male cadavers, which, consecutively, slightly changed the relations of the superior hypogastric plexus. After removing the posterior parietal peritoneum, we reached the aortic bifurcation and the initial portion of the common iliac arteries, as seen in Figure 1. Two groups of nerve fibers descended on the anterior surface of the abdominal aorta, originating in the prevertebral plexus, which merged immediately inferior to the aortic bifurcation and formed a relatively square nervous lamina, identified as the superior hypogastric plexus. This rectangular lamina had a length of about 4 cm to 5 cm and a width of about 2 cm. In our picture, we can observe thin branches from the superior hypogastric plexus, destined for the periarterial nerve plexus around the common iliac arteries. From the lower part of the plexus, the two hypogastric nerves emerge. A ureteric branch can also be seen arising from the right hypogastric nerve (following the principle that long organs have segmental vasculature and innervation from neighboring areas). In-depth dissection revealed the presacral vessels and the presacral fascia, as well as a retrorectal connective-adipose lamina extending between the two right and left hypogastric nerves.

**Figure 2:**
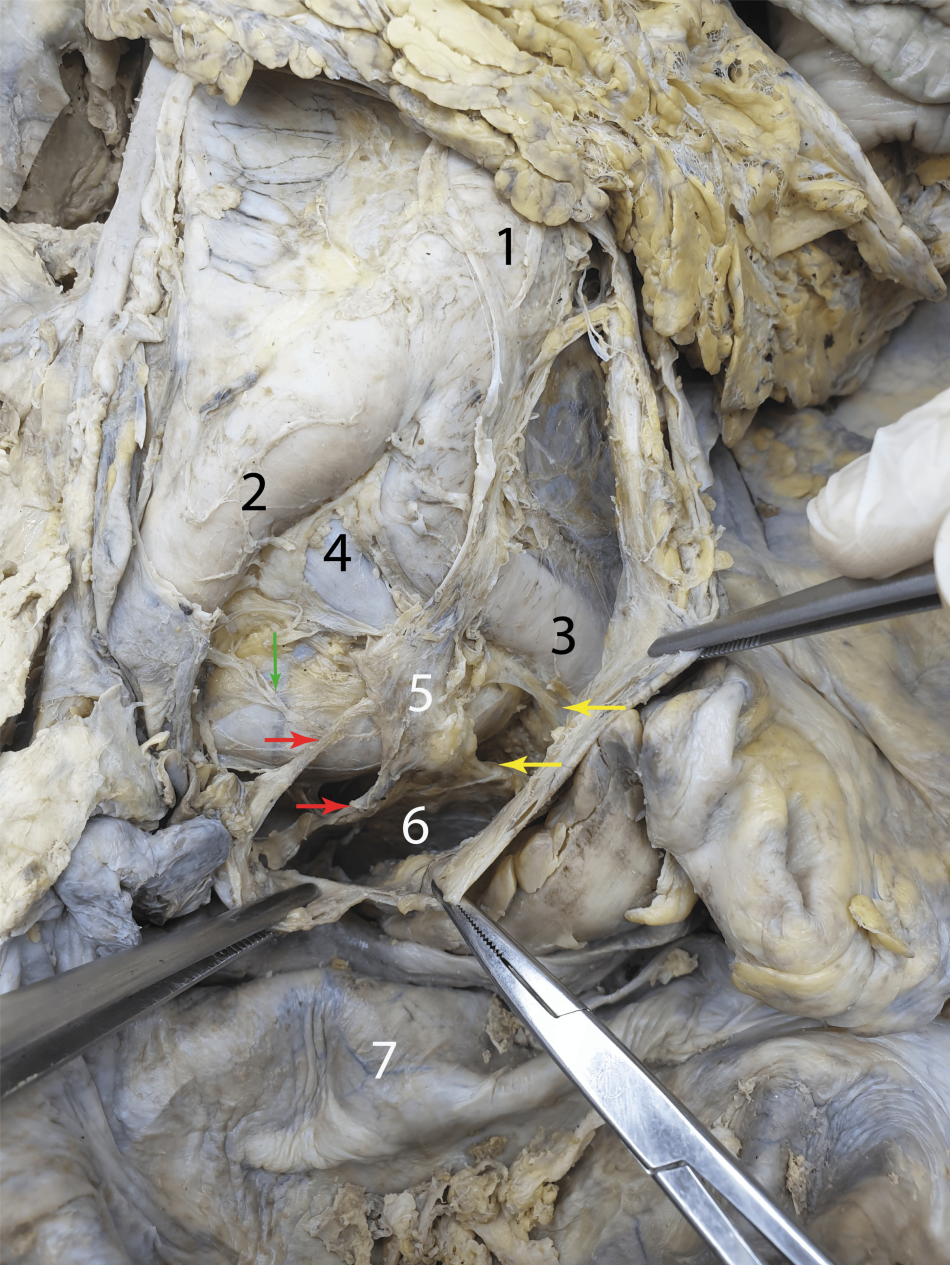
The relation of the superior hypogastric plexus after lifting the posterior parietal peritoneum The forceps and tweezers held the dissected posterior parietal peritoneum at the abdominopelvic border. Red arrows: Origin bundles of the right hypogastric nerve; Yellow arrows: Origin fascicles of the left hypogastric nerves; Green arrow: Arterial nervous branches for the common iliac artery; 1: Lumbar aorta, forming a convexity towards the left; 2: Right common iliac artery; 3: Left common iliac artery; 4: Left common iliac vein; 5: Inferior part of the superior hypogastric plexus; 6: Neuroconnective lamina between the hypogastric nerves; 7: The rectum tractioned anteriorly

Figure 2 shows an altered course of the lumbar aorta, which formed a convexity towards the left. This significant abnormality partially altered the relations of the superior hypogastric plexus. Although the plexus was still formed anterior to the lumbar aorta, slightly above the aortic bifurcation, and had the appearance of a nervous quadrangular lamina, it was slightly deviated to the left, so that it ended anterior to the left common iliac artery. At the lowermost point of the plexus, from its right side, the right hypogastric nerve was formed. The right hypogastric nerve intersected the left common iliac artery anteriorly and ended into a ganglionic lamina, from which branches emerged towards the left common iliac artery and the ureter. The left hypogastric nerve was also formed at the same level, mirroring the opposite one. The hypogastric nerves rapidly entered a subperitoneal route towards the pararectal peritoneal recesses. The left hypogastric nerve descended parallel to the superior border of the left common iliac artery and became subperitoneal at a greater distance from the rectum than on the right side. Part of the ganglionic lamina efferences described were contained within a retrorectal membranous neuroconnective lamina.

**Figure 3:**
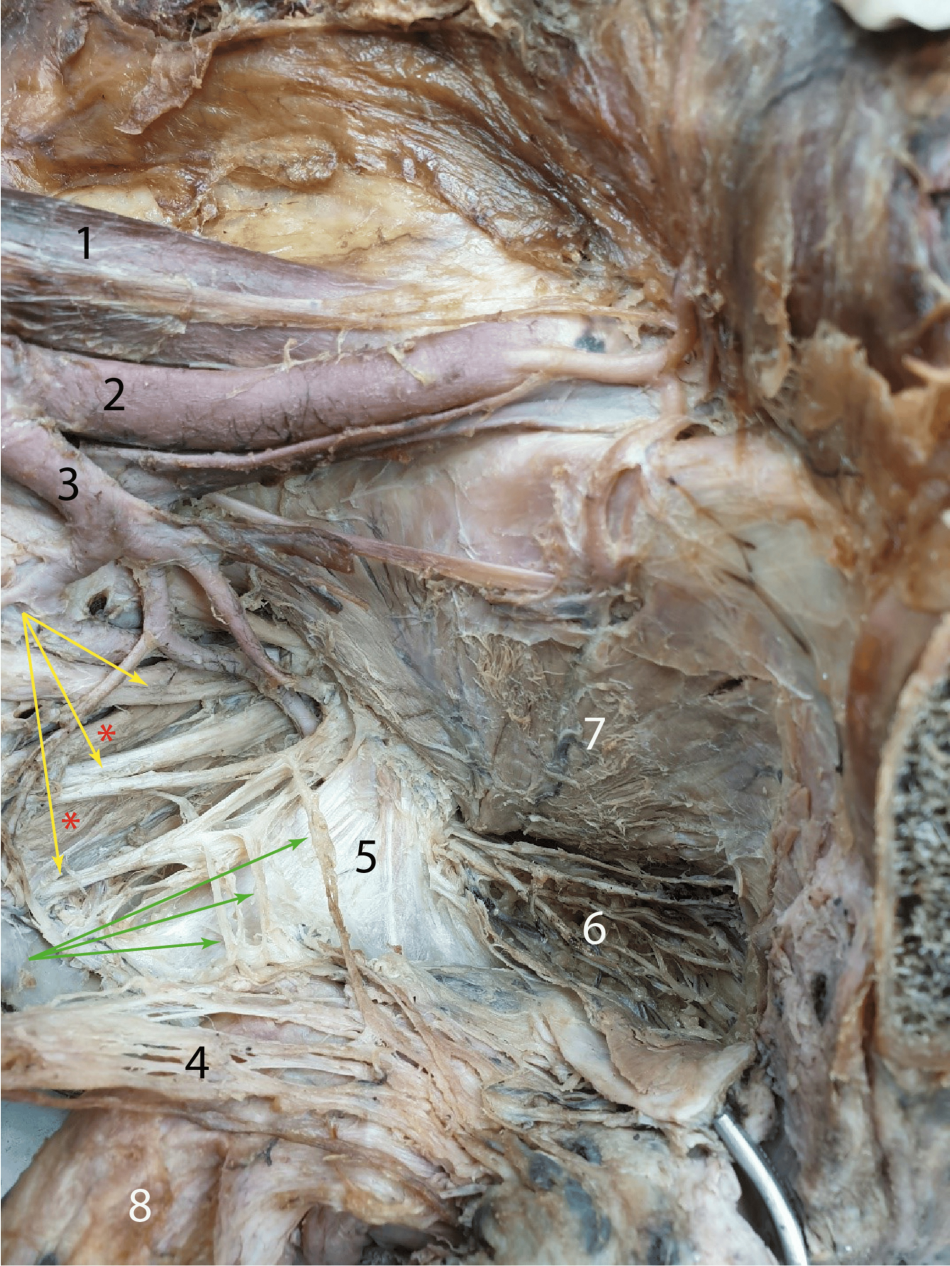
The erector nerves highlighted with numbers in the medial view of the left hemipelvis Yellow arrows: Sacral nerves S2, S3, S4; Green arrows: Pelvic splanchnic nerves; Red asterisks: Pyriformis muscle; 1: Iliopsoas muscle; 2: External iliac artery; 3: Internal iliac artery; 4: Inferior hypogastric plexus; 5: Sacrosciatic ligament; 6: Branches of the left pudendal nerve, visible after retracting the left levator ani muscle; 7: Internal obturator muscle; 8: The rectum tractioned in the medial view

Anterior to the sacrum, we highlighted the origin fascicles of the piriformis muscle, through which the anterior branches of the S2, S3, and S4 spinal nerves passed. These somatic nerve branches brought the parasympathetic motor fibers from the pelvic parasympathetic nucleus S2-S3-S4 into the pelvis. As can be seen in Figure 3, from the sacral spinal nerves, anastomotic branches towards the inferior hypogastric plexus emerged. These anastomoses are identified as Eckhardt's erector nerves, erigent nerves, pelvic splanchnic nerves, or erigent nerves. Parasympathetic fibers from the erector nerves took the path of the inferior hypogastric plexus, from which the cavernous nerve would arise.

**Figure 4:**
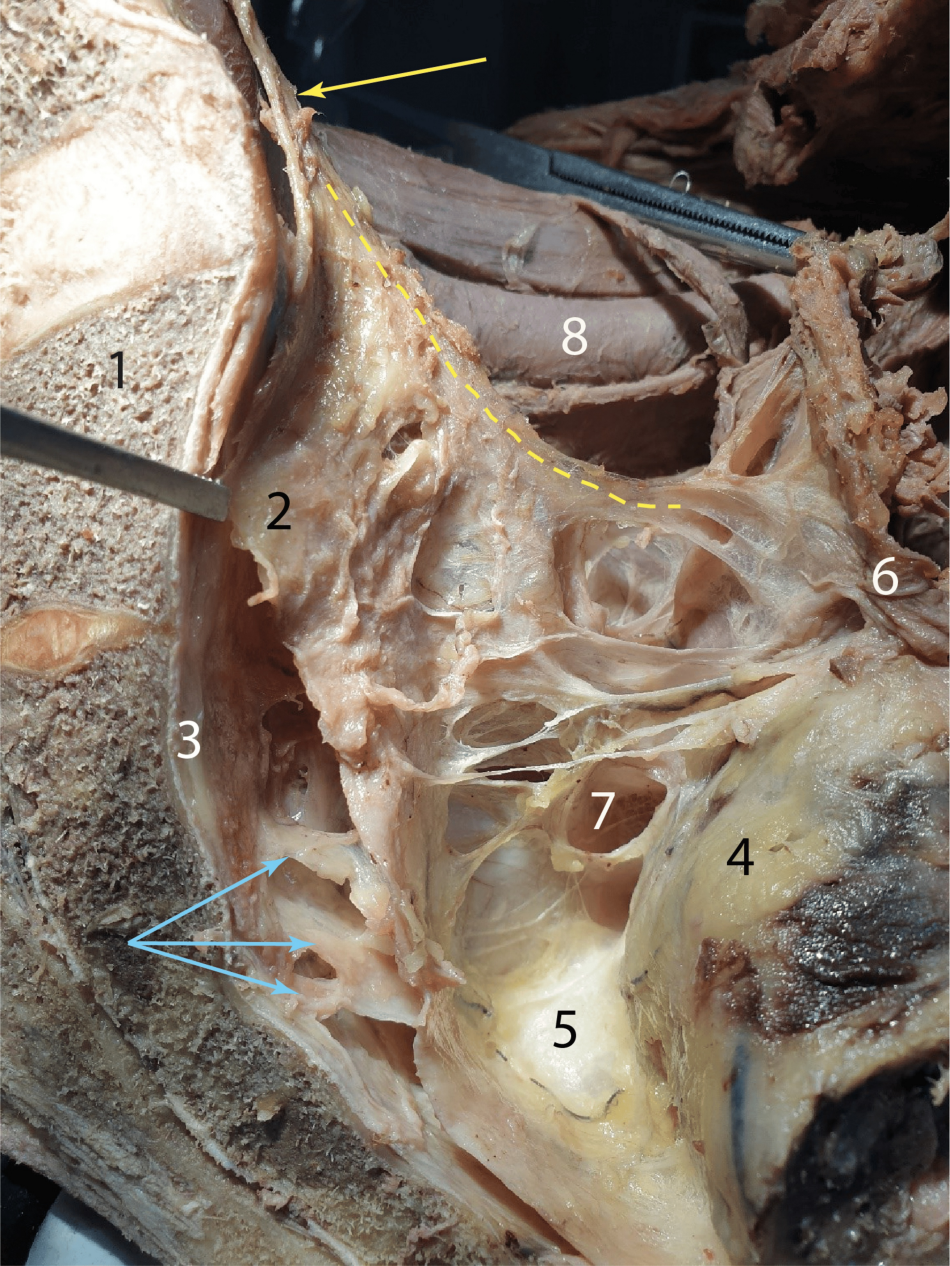
Sagittal section highlighting the appearance of the laterorectal subperitoneal structures in situ (dissection of the left hemipelvis) Yellow arrow: Left hypogastric nerve; Blue arrows: Erector nerves; Yellow dotted line: The pathway of the left hypogastric nerve found in close relation to the laterorectal peritoneum; 1: Sacrum; 2: Neuroconnective lamina between the hypogastric nerves; 3: Presacral fascia; 4: Rectum; 5: Angel's hair; 6: Posterior parietal peritoneum at the level of the left laterorectal recess; 7: Rectal nervous branches from the inferior hypogastric plexus; 8: Left external iliac artery

In Figure 4, the clamp contained the membranous connective lamina that connects the left and right hypogastric nerves retrorectally. The presacral fascia was found in direct relation to the sacrum, which in our picture was crossed by the erigent nerves S2 and S3. Posterior to the rectum, between it and the S3 and S4 vertebrae, was a mass of lax connective tissue, identified as the 'angel's hair'. This connective tissue was found in the dissection plane that allowed the separation of the rectum and the mesorectum from the sacrum. The left hypogastric nerve was held in tension and, therefore, suspended the laterorectal peritoneum, under which it engaged. In the pelvisubperitoneal space, the contours of the nerve fibers that comprise the superior hypogastric plexus can be distinguished. This dissection specimen has the merit of highlighting the exact risk area for sectioning the erigent nerves.

**Figure 5:**
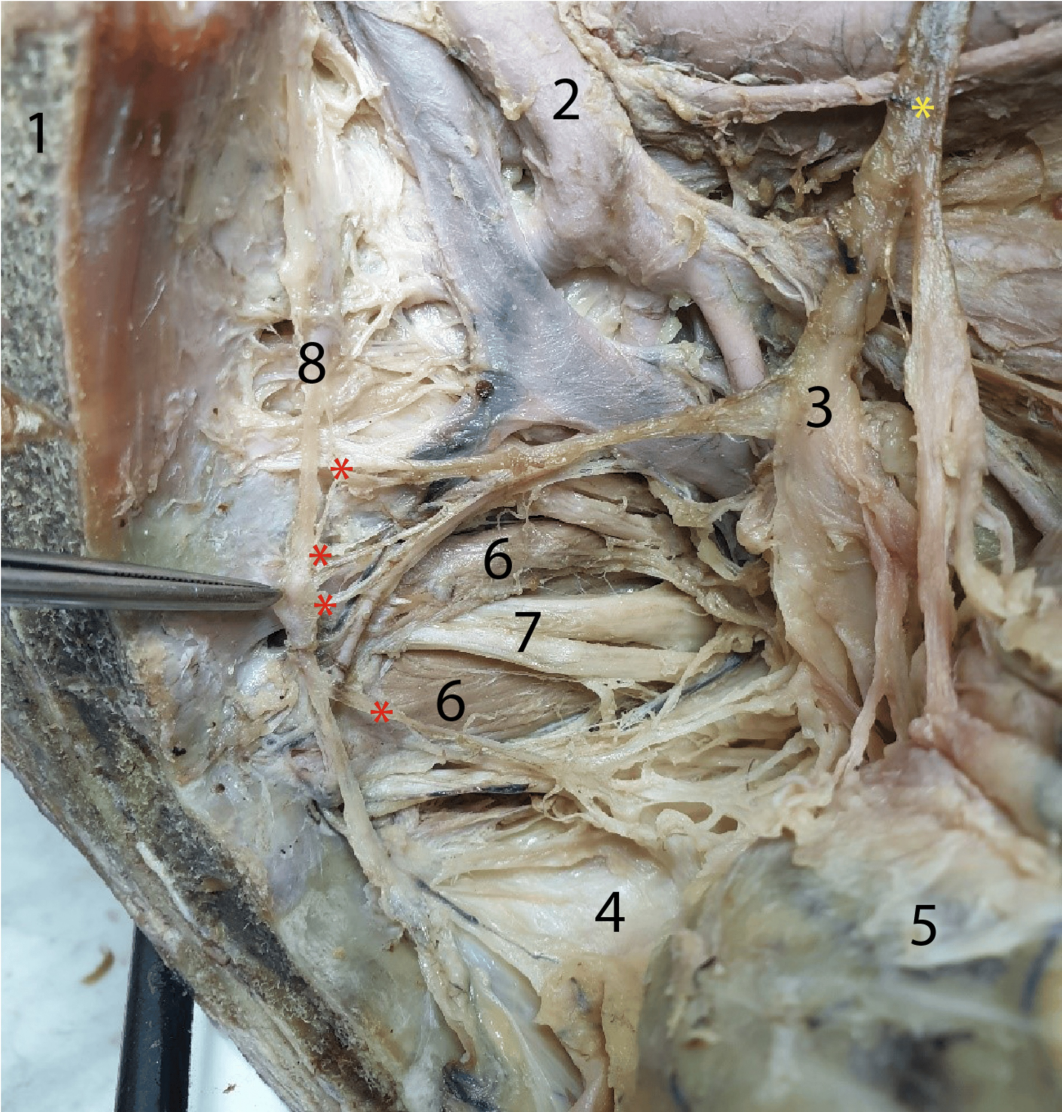
The sacral splanchnic nerves (sympathetic nerves) are highlighted with numbers in the dissection of the left hemipelvis, laterosacral region. Red asterisks: Sacral splanchnic nerves; Yellow asterisk: Left hypogastric nerve; 1: Sacrum; 2: Left internal iliac artery; 3: Left inferior hypogastric plexus; 4: Angel’s hair; 5: Rectum tractioned anteriorly; 6: Pyriformis muscle; 7: Branches of the sacral plexus; 8: Sacral sympathetic paravertebral chain

In Figure 5, we highlighted through dissection the nervous structures that carry the sympathetic impulses from the sacral sympathetic chain to the inferior hypogastric plexus. These nerves followed the path of the sacral sympathetic chain and, via the sacral splanchnic nerves, entered the inferior hypogastric plexus. Thus, we demonstrated that the sacral splanchnic nerves constitute anastomoses between the sacral sympathetic chain and the inferior hypogastric plexus. By detaching the left hypogastric nerve from the pelvic floor and drawing it superiorly, we highlighted the three nerve structures involved: the sacral sympathetic chain, the inferior hypogastric plexus, and the anastomoses between them, represented by the pelvic splanchnic nerves (Figure 5).

## Discussion

As we mentioned, modern surgery aims beyond the resection of the rectum and the mesorectum to both achieve oncological safety and preserve the patient's quality of life, performing the so-called nerve-sparing surgery [17]. This type of surgery refers to the preservation of the nervous structures that we highlighted through dissection to achieve a better postoperative quality of life. Furthermore, the implementation of minimally invasive techniques in surgery, corroborated with the use of high-definition cameras, has enabled surgeons to successfully recognize more anatomical structures, especially the nerve structures that are crucial to be protected. In pelvic surgical procedures, the principle of nerve-sparing surgery is mainly concerned with protecting the hypogastric nerves and the erector nerves, as well as the fibers of the inferior hypogastric plexus that carry the sympathetic and parasympathetic components towards the pelvic organs [18,19]. We managed to highlight important anatomical landmarks that allow the identification of these structures and their protection during surgical dissection.

The superior hypogastric plexus is retroperitoneal and in direct relation to the bifurcation of the aorta [20]. Sometimes, the superior hypogastric plexus is continued inferiorly with a ganglion-like lamina of different sizes and shapes (as seen in Figure 2). Also, the classical anatomical relations can be altered, as we demonstrated, for example, in the case of aortic kinking (the deviation of the normal course of the aorta, in this case in the lumbar part, also seen in Figure 2) [21]. Therefore, this major abnormality partially alters the relations of the superior hypogastric plexus, implying an additional anatomical risk in procedures such as the ligation of the inferior mesenteric artery, used in surgeries such as the anterior rectosigmoid resections or left hemicolectomies.

The right and left hypogastric nerves emerge from the lower part of the superior hypogastric plexus. They run inferiorly and laterally, in direct relation to the peritoneum of the pararectal recesses. This relationship is important for nerve protection in rectal resections [19].

The inferior hypogastric plexus originates from the abundant branching of the hypogastric nerve into the pararectal pelvisubperitoneal space [22,23]. This plexus originates mostly from the lumbar component of the sympathetic nervous system and provides sympathetic innervation for the pelvic organs. It also contains fibers from the sacral sympathetic chain and the sacral parasympathetic nerves [24].

The pelvic splanchnic nerves are anastomoses between the somatic spinal nerves of the sacral plexus S2-S3-S4, which also carry parasympathetic innervation, and the inferior hypogastric plexus [25,26]. These nerves are known as Eckhardt's erector nerves, pelvic splanchnic nerves, or erigent nerves. They carry parasympathetic vasodilatory impulses through the inferior hypogastric plexus towards the pelvic organs, i.e., the internal vesical and rectal sphincters and the genital erectile structures [27].

The sacral splanchnic nerves are anastomoses between the sacral sympathetic chain and the inferior hypogastric plexus. They carry sympathetic nerve impulses originating in the L1-L2 segments of the spinal cord to the pelvic organs [28]. They are represented by delicate, thin nerve structures that are difficult to see in direct relation to the pelvic wall. Consequently, they are more likely to be injured compared to the erector nerves.

The recognition and protection of pelvic nerves is of paramount importance for improving the quality of life of the patient. Notwithstanding that nerves have an essential role in pelvic organ function, the preservation of vessels in the deep, narrow pelvis also contributes to improved function. Therefore, surgeons should be familiar not only with emerging techniques for recognizing pelvic vessels, such as the use of indocyanine green but also with the nerve anatomy during pelvic surgeries to promote patients' safety [29].

Limitations

The number of dissected cadavers is a rather small one, in absolute terms. However, we believe that this constraint has been overcome by the particular quality of the dissection process, focused on the regional elements.

## Conclusions

The anatomical study through detailed dissections provides support for establishing modern surgical strategies in major abdominopelvic procedures, such as rectal resection surgeries. In our article, we have reviewed the whole set of nervous structures that need to be protected in rectal surgery, setting useful relations and optimal theoretical support for the surgical procedure. A thorough knowledge of the anatomical pathways and relations of the nervous structures that are located at or near the abdominopelvic border is crucial in the modern approach to local surgical and interventional procedures, especially in the case of pelvic-located cancers. These procedures focus not only on achieving oncological safety but also on maintaining the normal function of the pelvic organs. This aspect plays a significant role in the preservation of the quality of life in oncological surgery patients.
